# Policies to Support Lactation at Highly Ranked US Medical Schools

**DOI:** 10.1001/jamanetworkopen.2023.40048

**Published:** 2023-10-27

**Authors:** Lekshmi Santhosh, Ashley Vo, Caroline Wick, Michelle Mourad, Urmimala Sarkar, Reshma Jagsi, Christina Mangurian

**Affiliations:** 1 Division of Pulmonary/Critical Care Medicine, Department of Medicine, University of California, San Francisco; 2 University of California San Francisco School of Medicine, San Francisco; 3 University of California Campus Life Services, San Francisco; 4 Division of Hospital Medicine, University of California, San Francisco; 5 Center for Vulnerable Populations at Zuckerberg San Francisco General Hospital and Trauma Center, University of California, San Francisco; 6 Department of Radiation Oncology, Emory University, Atlanta, Georgia; 7 Department of Psychiatry and Behavioral Sciences, University of California, San Francisco

## Abstract

This cross-sectional study analyzes lactation support policies at the top 50 US schools of medicine.

## Introduction

Although the American Academy of Pediatrics recommends breastfeeding for at least 2 years,^1^ lactating physicians encounter multiple barriers preventing personal attainment of this goal.^2,3^ Physician leaders recently issued a call to action to support lactating physicians^4^; but to our knowledge, no studies have systematically examined the current state of lactation-support policies. This study aims to examine lactation-support policies at leading US schools of medicine (SOMs).

## Methods

This cross-sectional study follows the Strengthening the Reporting of Observational Studies in Epidemiology (STROBE) reporting guideline. Informed consent was not required because information was obtained from websites with no identifying information. This study was deemed exempt by the institutional review board at the University of California, San Francisco. From May through August 2022, we evaluated whether institutional lactation-support policies were present on publicly accessible websites for the top 50 US SOM per 2022 *US News and World Report* Rankings. We evaluated policies systematically for (1) dedicated space or equipment (ie, lists of lactation rooms, dedicated employee-only lactation rooms availability, pump availability); (2) accommodations (ie, clearly documented lactation policy, financial incentives to address the impact of lactation time on productivity, such as relative value unit [RVU] reimbursement or blocked clinic schedule slots, and supervisor toolkits); and (3) resources (ie, lactation consultants, peer or professional support, contact information for troubleshooting lactation room issues, and breastfeeding websites). Institution-level policies were obtained from each institution’s public website. Two authors verified policies by email with representatives from all 50 US SOMs. Descriptive statistics were conducted in Microsoft Excel and used to analyze the results.

## Results

In this cross-sectional study of 50 US SOMs, 47 SOMs (94%) had publicly available website information, and 50 (100%) had a clear list of lactation rooms and clearly posted institutional lactation policies. Additionally, 35 SOMs (70%) had employee-only lactation rooms and 25 (50%) provided pumps. Only 4 institutions (8%) provided financial incentives to make up for clinical time lost while lactating. Twenty-five SOMs (50%) provided toolkits for supervisors to support lactating employees returning from leave, 29 (58%) provided outpatient lactation consultant supports, and 23 (43%) provided peer support. Furthermore, 41 SOMs (82%) had an easily accessible contact if there were issues with lactation facilities, and 35 (70%) linked to websites about troubleshooting common lactation challenges. The Figure outlines the available services. Anonymized institution-specific details are noted in the Table.

**Figure.**
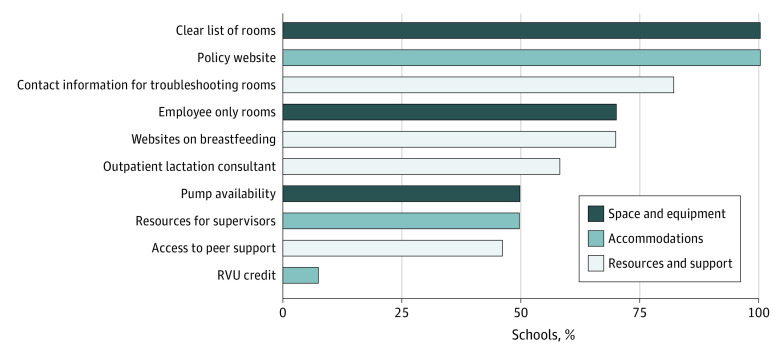
Lactation-Support Services Provided by Top 50 US Schools of Medicine (N = 50) RVU indicates relative value unit.

**Table.** Deidentified and Randomized List of 50 US Schools of Medicine and Their Lactation Policies and Accomodations

Institution	Clear list of rooms	Public room vs employee only	Support No.	Outpatient LC	Checklist for supervisors	Peer support	RVU credit	Websites?	Pumps?	Storage/refrigeration
1	Yes	Employee-only rooms provided	No	Yes, workshops	Yes	Yes	No	Yes	Yes	Yes (some)
2	Yes	No	No	Yes	No	Yes	No	No	No	Yes
3	Yes	Employee available	Yes	Yes	No	No	No	No	Yes	Yes
4	Yes	No	No	Yes, parenting assistance line available	No	Yes	No	Yes	No	Yes
5	Yes	Employee only	Yes	No	No	No	No	No	Yes	No
6	Yes	Faculty, student	Yes	No	Yes	No	No	No	No	Yes
7	Yes	Employee only	Yes	No	Yes	No	No	No	Yes	Yes
8	Yes	Public	Yes	Yes	Yes	No	No	Yes	No	Yes
9	Yes	Both available	Yes	Yes	Yes	Yes	No	Yes	Yes, discounted	Yes
10	Yes	NA	Yes	Yes	No	Yes	No	No	No	No
11	Yes	Employee only available	Yes	Yes	Yes	Yes	Yes	Yes	Yes	Yes
12	Yes	Employee only available	Yes	Yes	Yes	Yes	No	Yes	Yes	Yes
13	Yes	Employee only available	Yes	Yes	Yes	Yes	Yes	Yes	Yes	Yes
14	Yes	Employee only available	Yes	No	No	No	No	Yes	No	No
15	Yes	Faculty, student	Yes	Yes	Yes	No	Yes	No	Yes	No
16	Yes	Employee only available	Yes	Yes	No	Yes	No	Yes	No	Yes
17	Yes	No	No	No	No	No	No	No	No	No
18	Yes	Unclear if employee-only rooms are available	Yes	No	No	Yes	No	Yes	Yes	Yes
19	Yes	Yes	Yes	Yes	Yes	Yes	No	Yes	Yes	Yes
20	Yes	Employee only available	Yes	Yes, through employee insurance	Yes	No	No	Yes	Yes	Yes (some)
21	Yes	Employee only	Yes	No	No	Yes	No	Yes	No	Yes
22	Yes	No	No	No	Yes	No	No	No	Yes	No
23	Yes	Available to faculty, staff, and students only	Yes	No	Yes	Yes	No	Yes	No but kits	Yes
24	Yes	Employee only available and students	No	No	No	No	No	Yes	No	Yes (some)
25	Yes	Both?	Email	No	No	No	No	No	No but in process	Yes
26	Yes	Public?	Yes	No	Yes	Yes	No	Yes	Yes - some	Yes
27	Yes	Employee rooms (accessible to public with key access)	Yes	No	No	No	No	No	No	Yes
28	Yes	Public	No	No	No	No	No	No	No	Yes
29	Yes	Unclear if employee-only rooms are available	Yes	Yes	Yes	No	No	No	Yes	No
30	Yes	No	No	No	No	No	No	No	No	No
31	Yes	Public and employee only	Yes	Yes	No	Yes	No	Yes	?	Yes
32	Yes	No	Some rooms	Yes	No	No	No	App	Yes	Yes
33	Yes	Employee only available	Yes	Yes, via benefits	No, but in the process	No, but in process	No	Yes	No	No
34	Yes	Employee only	No	No	No	No	No	No	No	No
35	Yes	Employee only available	Yes	Yes	Yes	Yes	No	Yes	Yes	Yes
36	Yes	No	Yes	No, only with employee benefits	No	No	No	Yes	No	No
37	Yes	Public	Yes	Yes	Yes	Yes	No	No	No	No
38	Yes	Public	Yes	No	No	No	No	No	No	Yes
39	Yes	Employee only	No	No	No	No	No	No	No	Yes
40	Yes	Staff and students only	Yes	NA	Brief blurb only	No	No	No	No	Yes
41	Yes	NA	Yes	NA	NA	NA	NA	Yes	NA	Yes
42	Yes	No	Yes	Yes	Yes	Yes	No	Yes	Yes, but must bring own kit	Yes
43	Yes	No	Yes	Yes	No	No	No	Yes	Yes	Yes
44	Yes	Employee only	No	No	No	No	No	Yes	Yes	Yes
45	Yes	Public	Yes	Yes	Yes	Yes	No	Yes	No	Yes
46	Yes	Employee, staff, or student only	Yes	No	No	No	No	No	For rent	No
47	Yes	Public and resident-only	Yes	Yes	No	Yes	No	Yes	No	Yes
48	Yes	Employee only available	No	No	No	No	No	No	No	No
49	Yes	Employee only available	Yes	Yes	Yes	Yes	NA	Yes	Yes	Yes
50	Yes	Employee only	Yes	NA	NA	Yes	NA	Yes	NA	Yes

Abbreviations: LC, lactation consultant; NA, not available; RVU, relative value units.

## Discussion

All 50 SOMs had lactation-support policies that were available online and lists of lactation rooms. Although this is reassuring in light of the Patient Protection and Affordable Care Act, which requires employers to provide employees with adequate space and break time to express breastmilk for 1 year,^5^ more substantial evidence of break-time accommodations was limited. For example, less than 10% of programs provided financial incentives to address the impact of lactation time on physician productivity.^6^ This means that lactating physicians must choose between clinical compensation or taking breaks to express breastmilk and ensure adequate supply.

This study has limitations. The study was limited to the top 50 US SOMs, which limits generalizability since less-resourced allopathic and osteopathic SOMs may have even fewer resources for policy development and implementation. Using data from websites is another limitation of this study because it is difficult to know whether or not a school chose to publicize this information. However, manually verifying the information with the schools’ representatives adds additional validity to the data. Finally, we did not focus on identifying differences between various hospitals or affiliate sites, nor on the differences between policies for various groups (students, residents, fellows, faculty, staff). Because faculty may have private offices, students and trainees may be particularly vulnerable and even more adversely affected by a lack of lactation support. Further study could focus on the particular challenges for lactating students and trainees and the impact of accommodations on the training experience and well-being of trainees.

Our work suggests that leading US SOMs vary in lactation accommodations, and the diffusion of responsibility across multiple departments may also be a barrier to creating a comprehensive program. A robust lactation accommodation program should ideally include: (1) extensive investments in structural supports such as protected space and equipment; (2) peer support ranging from informational website to lactation consultants; and (3) accommodations policies that are easily accessible for employees and their managers. Although financial support for loss in productivity for lactating physicians is ideal, it is also the most difficult to enact. Universities can demonstrate their commitment to retaining and recruiting childbearing physicians with concrete mechanisms to account for this lost financial productivity. To truly promote gender equity and reproductive justice in medicine, policies within the profession itself must be modernized to reflect the needs of lactating physicians.

## Notes

Supplement.
Data Sharing Statement
