# Supplementary material for: Policies to Support Lactation at Highly Ranked US Medical Schools
Source: JAMA Netw Open. 2023 Oct 27;6(10):e2340048. doi: 10.1001/jamanetworkopen.2023.40048 (PMC10611993; doi:10.1001/jamanetworkopen.2023.40048)
Supplement: Supplement. — Data Sharing Statement [file jamanetwopen-e2340048-s001.pdf]

Data Sharing Statement

Santhosh. Policies to Support Lactation at Highly Ranked US Medical Schools. *JAMA Netw Open*. Published October 27, 2023. doi:10.1001/jamanetworkopen.2023.40048

Data
Data available: No
